# HIV partner services in Kenya: a cost and budget impact analysis study

**DOI:** 10.1186/s12913-018-3530-y

**Published:** 2018-09-17

**Authors:** Peter Cherutich, Carey Farquhar, Beatrice Wamuti, Felix A. Otieno, Ann Ng’ang’a, Peter Maingi Mutiti, Paul Macharia, Betsy Sambai, David Bukusi, Carol Levin, for the aPS Study Group

**Affiliations:** 1 Ministry of Health, Afya House, Cathedral Road, P.O Box 30016-00100, Nairobi, Kenya; 2 Department of Global Health, University of Washington, Seattle, WA USA; 3 Department of Research and Training, Kenyatta National Hospital, Nairobi, Kenya; 4 Department of Epidemiology, University of Washington, Seattle, WA USA; 5 Department of Medicine, University of Washington, Seattle, WA USA

**Keywords:** Budget impact analysis, Cost, HIV assisted partner services, Kenya

## Abstract

**Background:**

The elicitation of contact information, notification and testing of sex partners of HIV infected patients (aPS), is an effective HIV testing strategy in low-income settings but may not necessarily be affordable. We applied WHO guidelines and the International Society for Pharmaco-economics and Outcomes Research (ISPOR) guidelines to conduct cost and budget impact analyses, respectively, of aPS compared to current practice of HIV testing services (HTS) in Kisumu County, Kenya.

**Methods:**

Using study data and time motion studies, we constructed an Excel-based tool to estimate costs and the budget impact of aPS. Cost data were collected from selected facilities in Kisumu County. We report the annual total and unit costs of HTS, incremental total and unit costs for aPS, and the budget impact of scaling up aPS over a 5-year horizon. We also considered a task-shifted scenario that used community health workers (CHWs) rather than facility based health workers and conducted sensitivity analyses assuming different rates of scale up of aPS.

**Results:**

The average unit costs for HIV testing among HIV-infected index clients was US$ 25.36 per client and US$ 17.86 per client using nurses and CHWs, respectively. The average incremental costs for providing enhanced aPS in Kisumu County were US$ 1,092,161 and US$ 753,547 per year, using nurses and CHWs, respectively. The average incremental cost of scaling up aPS over a five period was 45% higher when using nurses compared to using CHWs (US$ 5,460,837 and US$ 3,767,738 respectively). Over the five years, the upper-bound budget impact of nurse-model was US$ 1,767,863, 63% and 35% of which were accounted for by aPS costs and ART costs, respectively. The CHW model incurred an upper-bound incremental cost of US$ 1,258,854, which was 71.2% lower than the nurse-based model. The budget impact was sensitive to the level of aPS coverage and ranged from US$ 28,547 for 30% coverage using CHWs in 2014 to US$ 1,267,603 for 80% coverage using nurses in 2018.

**Conclusion:**

Scaling aPS using nurses has minimal budget impact but not cost-saving over a five-year period. Targeting aPS to newly-diagnosed index cases and task-shifting to community health workers is recommended.

**Electronic supplementary material:**

The online version of this article (10.1186/s12913-018-3530-y) contains supplementary material, which is available to authorized users.

## Background

Assisted partner services (aPS), the systematic elicitation of contact information, exposure notification and active locating of sex partners of HIV infected patients, is essential for HIV prevention. aPS increases rates of HIV testing, improves HIV case finding and improves linkage to HIV care [1]. Although aPS is feasible and effective in limited resource settings [2, 3], further scale up may be dependent on its demonstrated cost-effectiveness, and affordability. In Malawi, assisted partner notification services by health providers were cost-effective compared to patient referral alone. However, this study was localized in an urban area and was clinic-based. [4]. Additional recent data from Kenya demonstrated that aPS is cost-effective with the incremental cost-effectiveness ratio per disability-adjusted life years averted being $1568 for nurses and $1156 for a task shifted approach to community health workers (CHW) [5]. No study has evaluated the budget impact of aPS in routine program settings in sub-Saharan Africa.

Budget impact analyses (BIA) are used alongside cost-effectiveness analyses to estimate the incremental impact of the intervention on affordability. The size of the eligible population is considered in the determination of BIA. Furthermore the current intervention mix, the expected mix and rate of scale up after the introduction of the new strategy are described [6]. Even if an intervention is highly cost-effective, donors may not fund it due to the size of the population in need of the intervention. It is increasingly likely that with the flat-lining of US Presidents Emergency Fund for AIDS Relief (PEPFAR) funding and the focus on the most cost-effective and targeted interventions, budget impact may become a requirement for funding as is the case in Europe and in the United States [7].

Kenya has the fourth largest HIV epidemic in the world with an estimated 600,000 HIV-infected persons who are unaware of their infection [8, 9]. This epidemic is larger in Western Kenya, including Kisumu County, which has the second highest prevalence of HIV in Kenya and is among the five counties contributing 51% of new HIV infections each year in Kenya [10]. The county is administratively autonomous, has a well-established HIV testing services (HTS) public health program and aPS would be considered a priority intervention, given that PEPFAR and the Kenya Ministry of Health (MoH) has already scaled down inefficient HTS strategies such as Voluntary Counseling and Testing (VCT) and door to door testing [11]. The county would therefore require information on program costs and a BIA to assess affordability of aPS as part of the HTS package. Currently, the HTS package includes HIV testing of patients either at health facilities or in communities and comprises minimal counselling support to enable HIV infected clients to disclose their HIV status to their sex partners. aPS would be added to HTS programs by active engagement of the HIV-infected patients and active tracing of their sex partners. Furthermore, implementing partners may choose to reduce program costs by utilizing task-shifting to lower cadres of health providers as is the norm in many public health settings.

In order to inform scale up of aPS in Kenya, we conducted a cost and budget impact analysis as part of a larger National Institutes for Health funded impact assessment of aPS delivered through existing HTS services in Kisumu county [12]. Overall, we assumed that the standard of care in Kenya is HTS and low-scale aPS at 5% coverage, and the intervention as aPS (HTS for index case, elicitation of sex partner information, tracing and testing of sex partners) delivered progressively over a five year time horizon to reach coverage of 50%.

## Methods

### Study design

We applied the International Society for Pharmacoeconomics and Outcomes Research (ISPOR) framework to perform the BIA [6]. Using a payer perspective, we constructed an Excel-based static deterministic model to simulate the budget impact analysis of aPS on an annual basis, over a 5 year time horizon for prevalent and incident HIV cases in Kisumu County. We compared current practice of HIV testing and partner referral alone to aPS based on HIV testing of index cases, locating their sex partners and HIV testing of these sex partners. We evaluate two scenarios for enhanced aPS services where the first scenario uses nurses and the second uses CHWs as part of primary health care.

### Study setting

The study setting was Kisumu county and we analyzed the BIA for Kisumu county’s health system that includes 9 hospitals, 20 health centres and 80 dispensaries that offer HTS. We anticipated that aPS would be offered to clients seeking HIV testing services in these health facilities and would be added to the existing Kenya MoH HTS services at three levels of the health systems: hospital, health centre and dispensary.

### Input data and sources

The standard of care includes HTS and passive request for the HIV infected client to notify their partners and ask them to test. aPS on the other hand includes HTS and an active process of enumerating sex partners of HIV infected persons and making phone calls to make these partners come for testing or visiting these partners at their homes or workplaces to test them*.* We therefore calculated costs for aPS which included costs for HTS for the HIV-infected index and their sex partner(s). Specifically, these costs included time for testing and counseling, elicitation and recording of partner information as well as for informing sex partners of their potential exposure to HIV. The costs also included airtime for contacting sex partners by phone and transport for locating them at their homes or places of work. Further to this, we determined costs that would accrue as a result of aPS (ART and clinic visits (Additional file 2). For all calculations we used an exchange rate of 87.70 Kenya shillings to one United States dollar.

#### Total aPS costs

*aPS* cost data were collected from a time in motion study and supplemented by additional cost data from study records and available market prices [5]. Cost categories were divided into mutually exclusive inputs and activities that included personnel, transportation, equipment, supplies, buildings and overhead, start up, recurring meetings, and data capture and use. We allocated the cost categories according to the WHO training manual on cost data collection for primary health care services [13].

The costs of rapid HIV test kits and consumable supplies were obtained from PEPFAR, and the Global Fund indicative prices. Fixed costs included program management (planning, administration, and supervision), staff training, travel, facility space, and equipment. These costs were annuitized over their useful lives. Recurrent program costs (personnel, planning, supervision and management) were estimated using local salary scales (Additional file 2).

Costs related to program start-up and staff training were estimated using the aPS study records. However we made adjustments for initial program supervision based on local per diem rates. Personnel time for HIV testing of index case and the sex partner was calculated based on staffing norms and full time equivalent and the possible number of HIV tests per staff per day. The case load per day was determined from previous studies and was within the national HTS standards which propose no more than 10 clients per day per counselor [12, 14, 15]. However, staff time for partner tracing and other provider related costs were collected using time and motion studies and was validated using expert opinion [5].

#### Unit costs for HIV testing

To estimate the unit cost for the current intervention mix we identified the total program costs for Kisumu county and divided these by the patient workload using the approach by Metlzer [16]. We assumed that dispensaries, health centres, and hospitals would have 2, 5 and 10 HIV positive patients per five-day week respectively and effectively constitute the upper bound for HIV testing costs. Conversely, we assumed 15, 25 and 50 HIV negatives per five-day week at dispensary level, health centre and hospital respectively, and this would constitute the lower bound for HTS costs. These numbers were based on previous formative research and reflect realistic volume load and existing national HTS guidelines [12, 14]. The unit cost for each HTS strategy was weighted by the annual HTS client load. We assumed that testing would follow the national algorithm which uses rapid HIV test kits, with minimal centralized lab testing [15]. As per the algorithm, all participants (index cases and their sex partners) were screened using a rapid HIV testing kit and if non-reactive were classified as HIV-negative. For those testing reactive on the screening test, they were subjected to confirmatory HIV rapid testing kit and if reactive on this second test were classified as HIV-positive. If non-reactive on this second test, the participant was referred to a central laboratory for further testing; however this number was minimal and not considered in the costing exercise.

#### Unit costs for aPS

The cost of aPS was determined as the aggregate costs of testing the index case, tracing their sex partner and testing the sex partner. The upper bound costs assumed that all index patients were HIV positive and all sex partners were HIV positive. The lower bound costs on the other hand were based on similar assumptions only that the sex partner would be HIV negative. In the aforementioned study, we recruited 1119 HIV-infected index cases who mentioned 1872 sex partners [1]. The cost of locating the sex partner was therefore a function of the number of sex partners mentioned per HIV-infected index patient (1.67) (1872/1119). Furthermore, since we located and enrolled 1305 sex partners and of these 787 consented to HIV testing, the cost of testing these partners per index case was weighted by 0.70 (787/1119) [1].

$$ \mathrm{Lower}\ \mathrm{bound}=\left(\mathrm{Cost}\ \mathrm{of}\ \mathrm{HIV}\ \mathrm{positive}\ \mathrm{index}\right)+\left({1.67}^{\ast}\mathrm{Cost}\ \mathrm{of}\ \mathrm{Locating}\ \mathrm{sex}\ \mathrm{partner}\right)+\left({0.70}^{\ast}\mathrm{Cost}\ \mathrm{of}\ \mathrm{HIV}\ \mathrm{negative}\ \mathrm{sex}\ \mathrm{partner}\right) $$

$$ \mathrm{Upper}\ \mathrm{bound}=\left(\mathrm{Cost}\ \mathrm{of}\ \mathrm{HIV}\ \mathrm{positive}\ \mathrm{index}\right)+\left({1.67}^{\ast}\mathrm{Cost}\ \mathrm{of}\ \mathrm{Locating}\ \mathrm{sex}\ \mathrm{partner}\right)+\left({0.70}^{\ast}\mathrm{Cost}\ \mathrm{of}\ \mathrm{HIV}\ \mathrm{positive}\ \mathrm{sex}\ \mathrm{partner}\right) $$

Across time, we assumed that the unit cost of aPS will rise as a function of scale up of aPS and marginally so, due to changes in eligible populations brought about by HIV transmission dynamics and survival (see Additional file 1).

### Eligible population

**Fig. 1:**
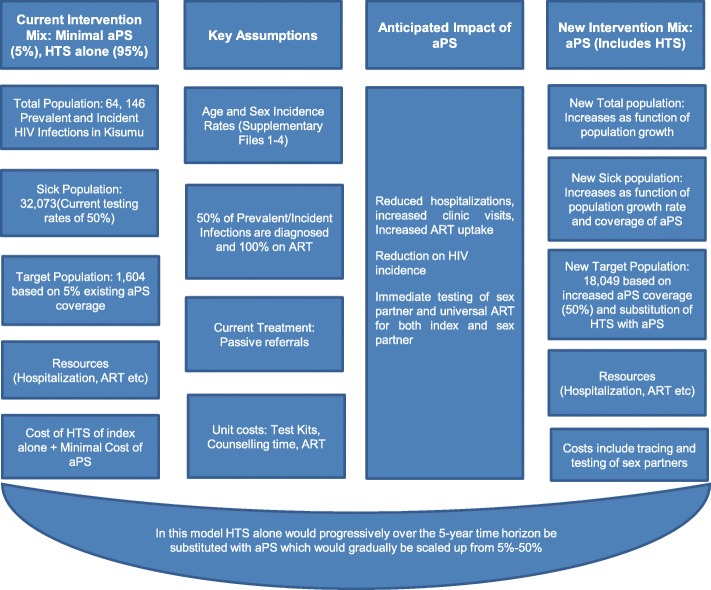
Schematic showing the elements of budget impact for aPS

The primary population for this intervention was prevalent and incident HIV cases in Kisumu county as estimated by the Kenya AIDS Indicator Survey 2012 and from UNAIDS Reference Group Estimates Spectrum model [8]. We anticipated that 50% of this population would be identified based on current rates of HIV testing [17, 18]. Furthermore, the size of this eligible population would rise marginally at a rate of 3% over the time horizon as a function of increased population growth rate, increased rate of HIV testing and increased life expectancy (Table 1, Fig. 1). We estimated little inward and outward migration. We assumed that aPS (HIV testing, tracing of partners and linkage to care) would progressively replace the standard of care to reach equilibrium at 50% coverage of all tests after five years of scale up. Specifically we modelled changes in eligible populations based on the intervention mix with 1604 receiving the expected aPS mix in 2014 to 18,049 receiving the same aPS mix in 2018.

**Table 1:** Eligible population, unit cost of aPS and 5-year coverage mix of aPS and standard of care, US$ 2014^§^

Year	Intervention Mix (Scale Up Rate)	Eligible Population	Nurse-Based Approach				CHW-Based Approach			
			Lower Bound		Upper Bound		Lower Bound		Upper Bound	
			Unit Cost	Total Cost	Unit Cost	Total Cost	Unit Cost	Total Cost	Unit Cost	Total Cost
2014	Standard of Care Mix (95%)	30,469.55	25.36	772,707.79	25.36	772,707.79	17.86	544,186.16	17.86	544,186.16
	aPS Mix(5%)	1603.66	44.75	71,763.79	53.07	85,106.24	32.04	51,381.27	33.72	54,075.42
	Total (100%)	32,073.21		844,471.57		857,814.02		595,567.43		598,261.58
2015	Standard of Care Mix (90%)	29,731.87	25.36	754,000.22	25.36	754,000.22	17.86	531,011.20	17.86	531,011.20
	aPS Mix(10%)	3303.54	44.75	147,833.42	53.07	175,318.87	32.04	105,845.42	33.72	111,395.37
	Total (100%)	33,035.41		901,833.64		929,319.09		636,856.62		642,406.57
2016	Standard of Care Mix (80%)	27,221.17	25.36	690,328.87	25.36	690,328.87	17.86	486,170.10	17.86	486,170.10
	aPS Mix(20%)	6805.29	44.75	304,536.73	53.07	361,156.74	32.04	218,041.49	33.72	229,474.38
	Total (100%)	34,026.47		994,865.60		1,051,485.61		704,211.59		715,644.48
2017	Standard of Care Mix (70%)	24,533.08	25.36	622,158.91	25.36	622,158.91	17.86	438,160.81	17.86	438,160.81
	aPS Mix(30%)	10,514.18	44.75	470,509.56	53.07	557,987.53	32.04	336,874.33	33.72	354,538.15
	Total (100%)	35,047.26		1,092,668.46		1,180,146.44		775,035.14		792,698.96
2018	Standard of Care Mix (50%)	18,049.34	25.36	457,731.26	25.36	457,731.26	17.86	322,361.21	17.86	322,361.21
	aPS Mix(50%)	18,049.34	44.75	807,707.97	53.07	957,878.47	32.04	578,300.85	33.72	608,623.74
	Total (100%)	36,098.68		1,265,439.23		1,415,609.74		900,662.07		930,984.96
Five-Year Total				5,099,278.50		5,434,374.90		3,612,332.84		3,679,996.54

^§^ *Weighted estimates of costs across three levels of health care (dispensary, health centre and hospital). HTS: HIV Testing Services. CHW: Community Health Worker. Upper Bound: If all sex partners are HIV positive*

*Standard of Care Mix: The unit cost is that of testing an HIV-infected index patient ONLY*

*aPS Mix: Sum of the unit cost of testing an HIV-infected index case PLUS per-index unit cost of tracing their sex partner PLUS per-index cost of testing the sex partner*

*Eligible Population increases annually as a function of population growth*

### Budget impact model

In the model we considered the following incremental costs: aPS, ART, clinic visits, and hospitalization (Fig. 1). ART costs were obtained from the Clinton Health Access Initiative indicative prices while costs related to hospitalization were obtained from published literature (Additional file 2). We parameterized the model to simulate increasing coverage of aPS from 5, 10, 20, 30 and 50% from year 1 to 5, respectively (Additional files 1). aPS roll out was assumed to increase ART-related costs including clinic visits, but avert hospitalizations. We assumed that patients would be linked to HIV care and start ART immediately upon diagnosis and stay on treatment for the time horizon with negligible drop-out rates. The costs of clinic visits were considered independent of the severity of HIV infection and the scale up strategy and assumed only to be associated with the coverage of aPS over the 5 year period. Costs related to hospitalization were assumed to accrue immediately. However we considered a lag of one year for costs of averted HIV infections to accrue in the standard of care scenario.

### Cost and BIA metrics

We present estimates of the lower and upper bound undiscounted total incremental HTS program costs over five years and the incremental cost needed to identify one HIV infected partner. Furthermore we report the proportional contribution to the budget impact of the various cost categories. These costs are reported separately for each eligible population and the program strategy (nurse-based or CHW-based). We also report the incremental budget impact of aPS for each year of scale up, within a five year budget cycle. These outcomes are separately reported for a nurse-based provider model and a task shift model using community health workers (CHWs). We assumed that patient outcomes were similar between the two cadres of health care workers.

All costs and BIA metrics were reported in 2014 US dollars for a single 12-month period and hence were not discounted. For the projected five year period, we used undiscounted costs.

### Sensitivity analyses

We explored various scenarios and conducted univariate deterministic sensitivity analysis of uptake of aPS ranging from 30 to 80% for the years 2014–2018 using both types of providers. We further provided for less than optimal linkage to ART for HIV-infected index cases and their sex partners, and calculated the budget impact for 90% and 100% linkage to ART. We assumed that HIV test kits and ART costs would vary little over the time horizon and did not include them in our sensitivity analyses.

We report costs of HIV testing and the annual and five-year financial consequences of HIV aPS in 2014 US dollars from a payer’s perspective. We also explore the costs and budget impact of a task shifting approach to aPS (see Additional file 1).

## Results

The total cost of HIV testing per index client varied across the three levels of health facilities, and by HIV status of sex partners of index cases. Differences in costs across the three levels of care were mainly driven by HTS client load and staff patterns, with higher level facilities performing more tests with relatively fewer staff. In the nurse-based model, this ranged from US$ 8.8 per index client assuming all HIV tests in a hospital were negative to US$ 100.8 per index client, if all HIV tests conducted in the lowest level clinic were positive. Using CHWs, the cost of a similar HIV test was US$ 5.6 per client when performed in a hospital for HIV-negative patients and US$ 62.8 per client for an HIV-infected case when conducted in the lowest level clinic. Table 2 presents the average unit costs of HIV testing across the three levels of facilities for index clients. Unit costs using nurses were US$ 25.4 and US$ 17.9 per client when using CHWs. The most significant difference between nurses and CHWs for the overall cost of the intervention was in the personnel related costs which represented between 54 and 70% of total costs, depending on the task-shifting approach. Supplies (including rapid HIV test kits and office stationery), took up close to a fifth of all costs (17%). Other costs were minimal. The costs of testing sex partners of index cases followed a similar pattern (Table 3). However, these costs were higher overall, with an HIV test costing US$ 19.2 if all tests were negative and US$ 311 if all tests were positive when conducted by nurses and US$ 11.7 and US$ 14.1 when given by CHWs. Upper-bound costs were 62.0% higher among nurses and 20.4% higher among CHWs. The cost of tracing a sex partner was US$3.57 and did not differ by the HIV status of the sex partners.

**Table 2:** Total and unit HTS costs for index case, per type of health provider and HIV-infection status, US$ 2014^§^

	HTS by Nurses				HTS by CHWs			
	HIV-positive		HIV-negative		HIV-positive		HIV-negative	
	Total cost	Unit Cost	Total cost	Unit Cost	Total cost	Unit Cost	Total cost	Unit Cost
Startup costs	5116.24	0.15	5310.57	0.06	3460.98	0.12	3460.98	0.04
Scale up costs								
Personnel	591,126.27	18.04	605,544.59	7.02	282,238.86	9.76	282,238.86	3.41
Transportation	21,057.62	0.57	19,936.61	0.20	32,249.85	0.82	32,249.85	0.31
Equipment	2748.28	0.08	2824.53	0.03	386.35	0.01	386.35	0.00
Supplies	142,372.87	4.22	155,258.61	1.76	142,372.87	4.23	156,278.75	1.76
Buildings and overheads	42,307.87	1.44	50,079.82	0.62	42,307.87	2.05	42,307.87	0.62
Data capture	28,306.36	0.87	28,475.31	0.33	27,386.62	0.88	27,386.62	0.32
Total Cost	833,035.49	25.36	867,430.05	10.03	530,403.39	17.86	544,309.27	6.47

^§^ *Weighted estimates of costs across three levels of health care (dispensary, health centre and hospital). HTS: HIV Testing Services. CHW: Community Health Worker*

**Table 3:** Total and unit HTS costs for sexual partners of HIV-infected index case, per type of health provider, US$ 2014^§^

Budget Category	HTS by nurses				HTS by CHWs			
	Lower Bound		Upper Bound		Lower Bound		Upper Bound	
	Total cost	Unit Cost	Total cost	Unit Cost	Total cost	Unit Cost	Total cost	Unit Cost
Startup costs	5310.57	0.13	5116.24	0.20	3544.79	0.09	3460.98	0.09
Scale up costs								
Personnel	605,544.59	14.84	591,126.27	22.95	290,615.24	7.19	282,238.86	7.23
Transportation	19,936.61	0.53	21,057.62	0.84	30,543.36	0.81	32,249.85	0.86
Equipment	2824.53	0.07	2748.28	0.11	372.21	0.01	386.35	0.01
Supplies	78,302.36	1.82	110,163.32	4.26	74,221.83	1.83	164,313.14	4.20
Buildings and overheads	50,079.82	1.07	42,307.87	1.61	50,079.82	1.12	42,307.87	1.04
Data capture	28,475.31	0.71	28,306.36	1.10	27,386.62	0.69	27,386.62	0.70
Total Cost	790,473.79	19.18	800,825.95	31.07	476,763.86	11.74	552,343.66	14.14

^§^*Weighted estimates of costs across three levels of health care (dispensary, health centre and hospital). HTS: HIV Testing Services. CHW: Community Health Worker*. *Lower bound assumes all tests at any given time are HIV-negative and upper bound assumes all tests at any given time are HIV-positive. Includes costs for community tracing of sexual partners*

Using the formulae described above, the higher bound cost of aPS was calculated to be US$ 53.07 per client, while the lower bound was US$44.75 per client for nurse-based testing and US$ 33.72 and US$ 32.04 for CHW-based approach, respectively.

The lower-bound total cost of scaling up aPS over a five period applying task shifting to CHWs was US $3.6 million and the upper-bound using nurses was US $ 5.4 million (Table 1). Partner services costs using CHWs were between 29 and 32% lower than those of nurse-based aPS delivery strategies. The cost differences between the upper bound and lower bound approaches were 1.9% among CHWs and 6.6% among nurses, respectively.

We calculated that aPS would identity an additional 11.9% of HIV infections; consequently increasing ART, hospitalization and clinic visit costs by the same margin. Additionally, aPS would incur US$ 1.78 less per patient owing to averted HIV infections.

The overall 5-year budget impact ranged from US$ 1,094,577 to US$ 1,767,863 for nurses and from US$ 1,191,185 to US$ 1,258,848 for CHWs. The aPS and ART costs contributed nearly the total of the budget impact.

**Fig. 2:**
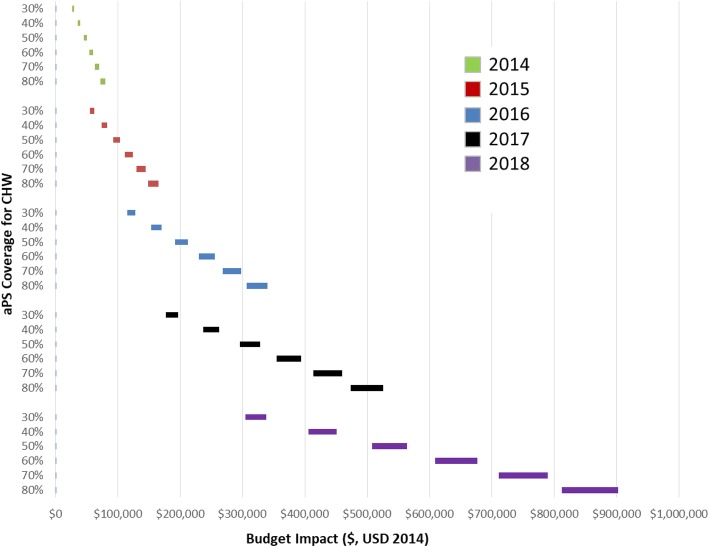
Sensitivity of budget impact to aPS coverage and linkage to ART for CHW-based aPS scale up, US$ 2014

**Fig. 3:**
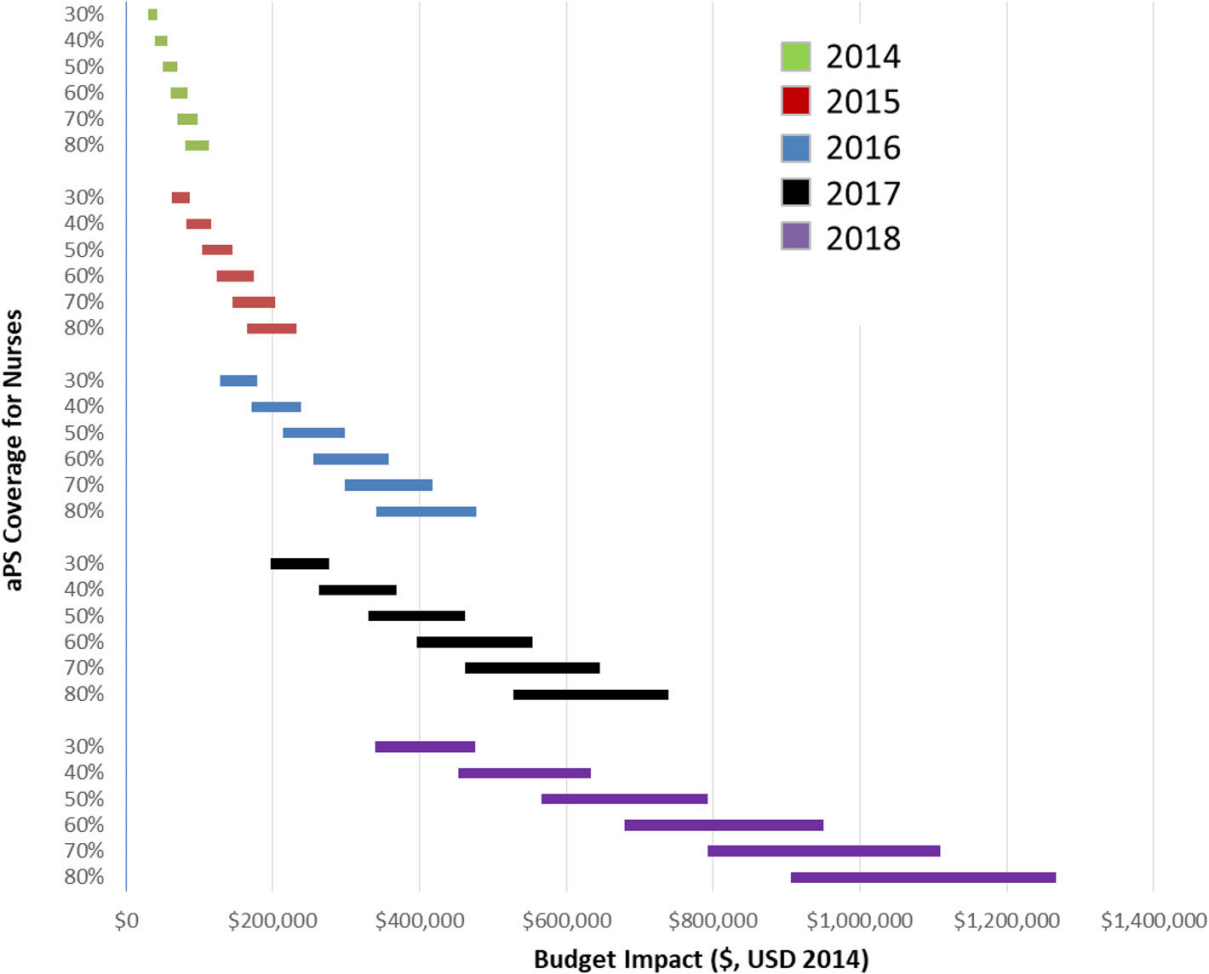
Sensitivity of budget impact to aPS coverage and linkage to ART for nurse-based aPS scale up, US$ 2014

In the sensitivity analysis and assuming different levels of aPS coverage in Kisumu county over a five year period, the budget impact varied across time, and between task shifting approaches. Compared to lower bound budget impact assuming 30% aPS coverage in 2014 using CHWs, the same upper bound impact in 2018 using nurses and assuming 80% aPS uptake was 4400% higher (US$ 1,267,603 versus US$ 28,547) (Table 4, Figs. 2 & 3). Taking into account rates of linkage to ART, the lower-bound impact for CHWs in 2014 assuming 30% coverage and 90% linkage to ART was US$ 27,060.80, while the upper-bound impact for nurses in 2018 assuming 80% coverage and 100% linkage to ART was US$ 1,267,603.30.

**Table 4:** Distribution of Budget Impact on Budget Categories over 5-year period, US$ 2014

	Nurse-Based Scale Up						CHW-Based Scale Up					
	Lower Bound			Upper Bound			Lower Bound			Upper Bound		
	Total	Per HIV Infected Index	% of Total Budget Impact	Total	Per HIV Infected Index	% of Total Budget Impact	Total	Per HIV Infected Index	% of Total Budget Impact	Total	Per HIV Infected Index	% of Total Budget Impact
aPS	646,959.42	0.07	0.59	1,116,048.32	0.11	0.63	571,113.86	0.06	0.48	638,777.57	0.06	0.51
ART	484,318.25	0.05	0.44	616,611.72	0.06	0.35	584,625.93	0.06	0.49	584,625.93	0.06	0.46
Clinic Visit	55,806.22	0.01	0.05	71,049.91	0.01	0.04	67,364.30	0.01	0.06	67,364.30	0.01	0.05
Hospitalization	(33,031.97)	(0.00)	(0.03)	39,873.25	0.00	0.02	39,873.25	0.00	0.03	39,873.25	0.00	0.03
Averted Infections	(59,474.76)	(0.01)	(0.05)	(75,720.53)	(0.01)	(0.04)	(71,792.64)	(0.01)	(0.06)	(71,792.64)	(0.01)	(0.06)
Total	1,094,577.16	0.12	1.00	1,767,862.68	0.18	1.00	1,191,184.71	0.12	1.00	1,258,848.41	0.13	1.00

## Discussion

We present the first data from an African setting of the financial consequences of scaling up aPS using HTS delivery settings. In Kisumu county, aPS would cost an additional US$ 11.4 M and US$ 7.5 M to existing HTS services when using nurses and CHWs, respectively, assuming all sex partners tested are HIV-infected. In financial year 2013/2014, the Kenyan government spent US$ 46 M for HTS services in Kenya, 43% of which came from PEPFAR [19]. In Kenya the policy to is to put resources in areas and among populations with the highest prevalence and incidence of HIV and Kisumu is considered a priority county on this account [10]. US$ 7.5 M which increases the budget by 16% would be considered a reasonable investment, particularly given the comprehensive aspect of aPS [19, 20]. In FY 2016, PEPFAR planned to spend US$ 30.6 M on HTS, and US$ 112.6 on ART [20].

Our results validate previous estimates of HIV testing. We determined that an HIV test costs US$ 10.03 per index client if all persons tested in a given year are HIV negative and US$ 25.03 per index client, if all are HIV-positive. In sub-Saharan Africa, an HIV test ranges from US$ 10–30 depending on the HIV testing algorithm, HTS strategy (community or facility) and methods and assumptions for cost calculation [21–23]. A positive HIV test costs more due to the requirement for a confirmatory test.

These findings differ substantially from those from other aPS costing studies. Our study estimated lower and upper bound values which closely reflect the dynamics of HIV testing within a program. True testing costs are stochastic values that depend on the background rates of HIV prevalence, intensity of case finding and staffing norms. Our estimates therefore may provide planners and program managers with the flexibility to weight HIV testing costs based on the local HIV prevalence and therefore budget more accurately. Importantly, our study assumed between 0.5 to 8 clients tested per day and this is within the workload recommendations in the national HIV testing standards [14, 23]. Our estimates therefore reflect the normative and practical patient workload for HTS.

As anticipated, a major driver of aPS unit costs, regardless of HIV status, was staff costs, specifically the health advisors, which is a new cadre in Kenya that is equivalent to a nurse. These costs will include hiring, training and retraining. Additionally, unit costs shall be affected by the added costs of community follow-up including transport and field allowance. These findings support task-shifting as a potential strategy for reducing costs for HIV testing and aPS in Africa. Community health workers reduce HIV testing costs by between 30 and 55% and overall aPS costs by an average of 30%. Additionally lower cadre workers improve health systems by reducing waiting times, reducing workload and enhancing quality of care [24].

In this study, over 90% of the budget impact was attributed to aPS and ART. Sustained efforts to reduce delivery costs of ART should be encouraged to include task-shifting of ART initiation and monitoring, point of care viral load measurements and lower drug prices. This would have significant impact on the scale up of aPS.

Not surprisingly, the budget impact was highly sensitive to the rate of scale up and this varied substantially over time. However, this impact was minimally sensitive to variations in linkage to ART. Even then, achieving coverage of 80% is still within the budgetary allocation of PEPFAR, which is the largest provider of HTS services in Kisumu county. Of note is that the total budget impact of aPS assuming 80% coverage is approximately US$ 360,000 lower using CHWs compared to nurses and this may present an attractive alternative to budget holders in the county. Utilization of CHWs for aPS could therefore be a realistic goal for national programs in Africa. This would be in line with the Kenya Community Health Strategy and builds on successful implementation of task shifting in Africa in HIV prevention, care and treatment [25, 26].

Our study has limitations. The static model applied did not fully capture the number and cost of infections that aPS would avert. While it may not be cost-saving, the time period we evaluated limited our ability to explore this. Furthermore, we did not capture the potential variation of ART and HIV test kit costs in the sensitivity analysis; neither did we perform a probabilistic sensitivity analysis. A wider range of linkage to care rates would have provided us with more insights on the sensitivity of linkage to care to the budget impact. The focus on coverage for the sensitivity analysis however is of particular interest to policy makers. Our estimates therefore provide policy makers with the appropriate framework to estimate resources required for aPS. Our approach is appropriate and is consistent with the ISPOR guidelines which prefer static models for shorter time horizons [6].

Given that aPS has been implemented in Kenya through the leadership of the first author and as a result of the findings from the main study, further studies would be required to validate our results.

## Conclusions

We conclude that aPS is effective, and has minimal budget impact and coupled with task shifting may present an opportunity to increase knowledge of HIV status in sub-Saharan Africa and lead to reduction of new HIV infections. To our knowledge this is the first budget impact analysis of aPS in Africa. As demonstrated, even with high levels of coverage, the overall budget impact of aPS is still within the PEPFAR’s HTS budgetary allocations for Kisumu county. Nevertheless, the impact was highly sensitive to the task-shifting approach and the level of uptake of the service. Greater impact would be achieved if aPS was targeted to newly diagnosed and ART-naïve index cases who present a high risk of HIV transmission.

## Additional files

Additional file 1: Cost and Budget Impact Model Parameters and their outputs. (ZIP 1380 kb)

Additional file 2: Appendix I: Sources of data and assumptions, for budget impact analysis of aPS, Kisumu County, US$ 2014. Appendix II: Sources of data and assumptions of costing of HIV testing and aPS, Kisumu County, US$ 2014. Appendix III: HIV testing case load per level of health facility. (DOCX 40 kb)

## Abbreviations

aPS: assisted partner services
ART: Antiretroviral therapy
BIA: Budget impact analysis
CHW: Community health workers
DALY: Disability-adjusted life years
HIV: Human immuno-deficiency syndrome
HTS: HIV testing services
ISPOR: International Society for Pharmacoeconomics and Outcomes Research
MoH: Ministry of Health
PEPFAR: US President’s Emergency Plan for AIDS Relief
UNAIDS: The Joint United Nations Programme on HIV/AIDS
VCT: Voluntary Counseling and Testing
WHO: World Health Organization
