# Supplementary material for: HIV partner services in Kenya: a cost and budget impact analysis study
Source: BMC Health Serv Res. 2018 Sep 17;18:721. doi: 10.1186/s12913-018-3530-y (PMC6142360; doi:10.1186/s12913-018-3530-y)
Supplement: Supplementary file 2 — Appendix I: Sources of data and assumptions, for budget impact analysis of aPS, Kisumu County, US$ 2014. Appendix II: Sources of data and assumptions of costing of HIV testing and aPS, Kisumu County, US$ 2014. Appendix III: HIV testing case load per level of health facility. (DOCX 40 kb) [file 12913_2018_3530_MOESM2_ESM.docx]

Appendices

Appendix I: Sources of data and assumptions, for budget impact analysis of aPS, Kisumu County, US$ 2014

| **Characteristics** | **Description** | **Number** | **Source of Data/Assumptions** |
| --- | --- | --- | --- |
| Eligible Population | All HIV-infected persons(15-64) | 118,538 | Spectrum/KAIS/UNAIDS/ Prevention Revolution Roadmap[^2^](#_ENREF_2). Modelled estimates |
| Eligible Population | All HIV infected persons on ART | 62,280 | Kenya HIV and AIDS Profile by County[^3^](#_ENREF_3). Modeled estimates |
| Coverage of HTS | Proportion of HIV-infected persons tested for HIV | 50% | Nganga et al, KAIS 2012[^4^](#_ENREF_4). National estimates apply to Kisumu county |
| Cost of aPS | Cost per index case | Nurse Lower Bound: US 49.73  Nurse Upper Bound: US$ 61.15  CHW Lower Bound: US$ 35.09  CHW Upper Bound: US$ 37.40 | aPS study: Upper bound: *Cost of HTS index case and cost of tracing and testing a HIV positive sexual partner*. Lower bound: *Cost of HTS index case and cost of tracing and testing a HIV negative sexual partner*  Average of 1.67 partners per index with 60% locatability and testing. The cost of tracing/locating sex partners was calculated at US$ 3.57 based on time in motion studies(Suppl_6_Weighted Costs.xlsx) |
| ART cost | Annual delivery cost per person | US$ 243 per annum | CHAI prices (US$ 250) Zambia Scott et al, 2014[^5^](#_ENREF_5) (US$ 243) Zambia. Actual based on tender prices  Zambia costs are applicable to Kenya. Costs include drugs, personnel and laboratory monitoring. We assume that ART would be initiated immediately after diagnosis and continued without any loss to follow-up. ART effiicay in averting infection was assumed at 96%. |
| Hospitalization | Reduction in probability of hospitalization | 49% | Meyer-Rath et al[^6^](#_ENREF_6). Immediate ART initiation is as effective |
| Hospitalization | Hospitalization rate  per 100 patient  years | 4.9 for Pre-ART patients  6.9 for ART patients | Meyer-Rath, et al, 2013[^6^](#_ENREF_6). |
| Hospitalization | Unit cost | US$ 72 for Pre-ART patients  US$117 for ART patients | Meyer-Rath, et al, 2013[^6^](#_ENREF_6) |
| HIV transmission probabilities | M: F and F: M Per coital HIV infectivity | M:F -0.0019  F:M-0.0010 | Hughes et al, 2012[^7^](#_ENREF_7). Apply the higher probability of 0.0019 |
| Averted HIV infections | Cost and efficacy of ART | Lifetime cost of ART-no genotype-US$ 16,360  Efficacy of ART-96% | Enns et al, Int Journal STDs 2011[^8^](#_ENREF_8). Levison et al, CID 2013[^9^](#_ENREF_9). Cohen et al, 2011[^10^](#_ENREF_10). aPS would avert infection through increase uptake of ART and reduction of concurrency |

Appendix II: Sources of data and assumptions of costing of HIV testing and aPS, Kisumu County, US$ 2014

| **Characteristics** | **Description** | **Number** | **Source of Data/Assumption** |
| --- | --- | --- | --- |
| HIV test kit prices | Unit prices per person | KHB( US$1.48)  First Response(US$1.94) | Global Fund, Kenya MoH. Actual based on tender prices |
| Personnel time (hrs) for community tracing of partners | Full time equivalent for health providers | 2 hrs per client | Time motion studies in the aPS study. Asymptotic validity is achieved. Same FTE regardless of cadre |
| Salaries for nurses and CHWs | *Median* annual salaries per cadre | Nurses(US$ 630) per month  CHWs(US$ 230) per month | Salaries & Remuneration Commission Kenya and aPS study records. Majority of nurses and CHW would be in job group F/G & G,H,J |
| Buildings | Cost per Surface area | US$2.00 per square feet | HASS consult price index[^1^](#_ENREF_1). Public sector buildings have same value as private sector’s |

Appendix III: HIV testing case load per level of health facility

| Health care Level | # days worked/ year | # HTS staff in all facilities | # tested^^ | # tested^§^ | Annual # tested^γ^ | | Annual # tested^€^ | HIV-Infected cases per facility | # Outpatient Visits per facility |
| --- | --- | --- | --- | --- | --- | --- | --- | --- | --- |
| Hospital | 215 | 58 | 8 | 3 | 37,410 | 99,760 | | 37,000 | 107,000 |
| Health Centre | 215 | 64 | 5 | 2 | 27,520 | 68,880 | | 20,000 | 60,000 |
| Dispensary | 215 | 128 | 3 | 0.5 | 13,760 | 82,560 | | 5,000 | 5,500 |

^^ Number tested per day if all patients are HIV negative (upper bound). ^§^ Number tested per day if all patients are HIV positive (lower bound). ^γ^ Annual Number tested(lower bound). ^€^ Annual Number tested (upper bound). The case load was derived from experience from the study and are within the norms of the Kenya national HTS standards (maximum 10 clients per day per counsellor).

1. http://www.hassconsult.co.ke/index.php?option=com_content&view=article&id=22&Itemid=29.

2. Programme NASC. Kenya HIV Prevention Revolution Roadmap. 2013.

3. Ministry of Health K. HIV and AIDS Profile by County. 2014.

4. Ng'ang'a A, Waruiru W, Ngare C, et al. The Status of HIV Testing and Counseling in Kenya: Results From a Nationally Representative Population-Based Survey. *Journal of acquired immune deficiency syndromes* 2014; **66 Suppl 1**: S27-36.

5. Scott CA, Iyer HS, McCoy K, et al. Retention in care, resource utilization, and costs for adults receiving antiretroviral therapy in Zambia: a retrospective cohort study. *BMC public health* 2014; **14**(296): 1471-2458.

6. Meyer-Rath G, Brennan AT, Fox MP, et al. Rates and cost of hospitalization before and after initiation of antiretroviral therapy in urban and rural settings in South Africa. *Journal of acquired immune deficiency syndromes* 2013; **62**(3): 322-8.

7. Hughes JP, Baeten JM, Lingappa JR, et al. Determinants of per-coital-act HIV-1 infectivity among African HIV-1-serodiscordant couples. *The Journal of infectious diseases* 2012; **205**(3): 358-65.

8. Enns EA, Brandeau ML, Igeme TK, Bendavid E. Assessing effectiveness and cost-effectiveness of concurrency reduction for HIV prevention. *International journal of STD & AIDS* 2011; **22**(10): 558-67.

9. Levison JH, Wood R, Scott CA, et al. The clinical and economic impact of genotype testing at first-line antiretroviral therapy failure for HIV-infected patients in South Africa. *Clinical infectious diseases : an official publication of the Infectious Diseases Society of America* 2013; **56**(4): 587-97.

10. Cohen MS, Chen YQ, McCauley M, et al. Prevention of HIV-1 infection with early antiretroviral therapy. *The New England journal of medicine* 2011; **365**(6): 493-505.
